# Polysaccharide of Atractylodes macrocephala Koidz alleviate LPS-induced inflammatory liver injury by reducing pyroptosis of macrophage via regulating LncRNA GAS5/miR-223-3p/NLRP3 axis

**DOI:** 10.3389/fphar.2025.1593689

**Published:** 2025-07-29

**Authors:** Xiaoxiao Chen, Shuzhan Yang, Xin Long, Wanyan Li, Bingxin Li, Cheng Fu, Caoxue Zhen, Danning Xu, Xinliang Fu, Nan Cao

**Affiliations:** ^1^College of Animal Science and Technology, Zhongkai University of Agriculture and Engineering, Guangzhou, China; ^2^Guangzhou Customs, Technology Center, Guangzhou, China

**Keywords:** pyroptosis, polysaccharide of Atractylodes macrocephala Koidz, liver inflammation, macrophage, ceRNA

## Abstract

**Introduction:**

Pyroptosis is a distinctive form of inflammatory cell death, mediated by the activation of the inflammasome, which initiates a potent inflammatory response. Long non-coding RNAs (lncRNAs) modulate pyroptosis by targeting microRNAs and their target genes through a competing endogenous RNA (ceRNA) mechanism. Our previous research has confirmed that Polysaccharide of Atractylodes macrocephala Koidz (PAMK) can alleviate inflammatory liver injury in mice caused by lipopolysaccharide (LPS), but the specific molecular mechanism remains unclear. Additionally, recent studies have identified recruited macrophages in the liver as a key component of both acute and chronic liver inflammation. This study aimed to explore the impact of PAMK on LPS-induced macrophage pyroptosis and its molecular mechanism in mitigating inflammatory liver injury in mice.

**Methods:**

C57BL/6 mice were subjected to LPS-induced liver injury with or without PAMK pretreatment. Histopathological analysis, qRT-PCR, Western blot, and ELISA were performed to assess liver damage and pyroptosis markers. In RAW264.7 macrophages, dual-luciferase assays validated ceRNA interactions, while gain/loss-of-function experiments elucidated molecular mechanisms.

**Results:**

In this study, we found that PAMK alleviated LPS-induced inflammatory liver injury in mice and modulates macrophage pyroptosis through the lncRNA GAS5/miR-223/NLRP3 axis.

**Conclusion:**

We conclude that there is a ceRNA relationship between GAS5, miR-223-3p, and NLRP3; PAMK alleviates LPS-induced pyroptosis in macrophages through the lncRNA GAS5/miR-223-3p/NLRP3 axis; and PAMK intervention in the macrophage pyroptosis process subsequently alleviates liver inflammation.

## 1 Introduction

Pyroptosis is a form of programmed cell death mediated by the gasdermin family proteins (Broz et al., 2020). In the canonical pathway, caspase-1 is recruited and activated by the inflammasome, resulting in the cleavage of gasdermin D (GSDMD). This cleavage generates the active N-terminal fragment of GSDMD (GSDMD-N), which translocates to the cell membrane and forms pores, leading to pyroptosis. NOD-like receptor thermal protein domain associated protein 3 (NLRP3) activation is involved in various liver diseases (Shi et al., 2020). Stimulation by LPS induces the activation of the NLRP3 inflammasome (Boaru et al., 2012). Activation of the NLRP3 inflammasome triggers the activation of caspase-1, which cleaves gasdermin family proteins and releases the N-terminal domain with pore-forming activity, resulting in cellular pyroptosis. This process subsequently leads to significant release of Interleukin 1 Beta (IL-1β) and Interleukin 18 (IL-18), exacerbating the inflammatory response (Cho et al., 2019). GAS5 is a long non-coding RNA (lncRNA) that has been reported to play a role in inflammation by affecting miRNA expression (Mo et al., 2022; Xu et al., 2020). MiR-223, as an anti-inflammatory regulator, can modulate gene expression by inhibiting mRNA translation and thus affect the inflammatory process (Bauernfeind et al., 2012; Huppertz et al., 2020). Studies have reported downregulation of miR-223 in LPS-induced injury models (Zhao et al., 2018). It has been shown that there is a ceRNA relationship between GAS5, miR-223, and NLRP3, which plays a role in neuroinflammation (Xu et al., 2020). However, the role of this relationship in liver inflammation remains unclear.

Polysaccharide of Atractylodes macrocephala Koidz (PAMK) are the major bioactive components of the botanical drug Atractylodis Macrocephalae. They possess various beneficial effects such as immune enhancement, growth promotion, and maintenance of body homeostasis. Studies have shown that PAMK can protect against liver injury caused by various factors. It has been demonstrated to regulate LPS-mediated hepatic inflammation in mice (Guo et al., 2021). Additionally, PAMK can alter the ceRNA relationship in animals and regulate liver inflammation through the miR-223/NLRP3 pathway (Chen F et al., 2023; Wu et al., 2022). The hepatoprotective effect of PAMK has been validated in different models of liver injury. However, the precise mechanism underlying the hepatoprotective effect of PAMK in liver inflammatory injury remains undefined.

Macrophages play a complex and pivotal role in the liver, being an indispensable ingredient of both acute and chronic liver inflammation (Zeng et al., 2021). Through mechanisms such as the degradation of the extracellular matrix, macrophages have a dual role in modulating the inflammatory response, capable of both alleviating and exacerbating inflammation (Sun et al., 2021). Furthermore, macrophages can release inflammatory cytokines, which recruit additional immune cells to the liver and may intensify or, in some cases, alleviate liver disease (Rao et al., 2022).

This study investigates the regulatory role of PAMK in LPS-induced inflammatory liver injury at the animal level, with C57BL/6 mice as the research subjects. It also explores the interaction mechanisms of GAS5, miR-223-3p, and NLRP3 in the regulation of LPS-induced inflammatory liver injury by PAMK at the cellular level, using RAW264.7 cells as the research objects. The aim is to provide accurate and detailed theoretical basis for the in-depth exploration of PAMK’s positive intervention in liver injury process.

## 2 Materials and methods

### 2.1 Source of drugs

Polysaccharide of Atractylodes macrocephala Koidz (PAMK, purity ≥95.0%) used in our study was purchased from Xi’an Tianyuan Biotechnology Co. PAMK was extracted from Atractylodis macrocephalae Rhizoma from Zhejiang Province, China. During the production process, the manufacturer implemented strict process parameters and quality inspection of the intermediates in key processes, such as properties and polysaccharide content. The purity, molecular weight and monosaccharide composition of all final products were investigated. The quality inspection reports offered by the manufacturer showed that there were few differences in the molecular weight and monosaccharide composition between different batches of PAMK, indicating the stable reproducibility of PAMK preparation.

### 2.2 Characterization of PAMK

Approximately 5 mg of PAMK was hydrolyzed with trifluoroacetic acid (2 M) at 121 C for 2 h in a sealed tube. Dry the sample with nitrogen. Add methanol to wash, then blow dry, repeat methanol wash 3 times. The residue was redissolved in deionized water and filtered through 0.22 μm microporous filtering film for measurement. The sample extracts were analyzed by high-performance anion-exchange chromatography (HPAEC) on a CarboPac PA-20 anion-exchange column (3 by 150 mm; Dionex) using a pulsed amperometric detector (PAD; Dionex ICS 5000^+^ system). Flow rate, 0.5 mL/min; injection volume, 5 μL; solvent system A: (ddH_2_O), solvent system B: (0.1 M NaOH), solvent system C: (0.1 M NaOH, 0.2 M NaAc); gradient program, volume ratio of solution A, B, C was 95:5:0 at 0 min, 85:5:10 at 26 min, 85:5:10 at 42 min, 60:0:40 at 42.1 min, 60:40:0 at 52 min, 95:5:0 at 52.1 min, 95:5:0 at 60 min. Data were acquired on the ICS5000^+^ (Thermo Scientific), and processed using Origin2019. We evaluated the monosaccharide composition and percentages using fucose (Fuc), rhamnose (Rha), arabinose (Ara), galactose (Gla), glucose (Glc), xylose (Xyl), mannose (Man), fructose (Fru), ribose (Rib), galacturonic acid (Gal-UA), glucuronic acid (Glc-UA), mannuronic acid (Man-UA) and guluronic acid (Gul-UA) as references.

### 2.3 Animals and treatments

One hundred 5-week-old female Balb/c mice (SPF grade) purchased from the Animal Center of Southern University of Science and Technology were randomly divided into 4 groups (n = 25 per group). Mice were placed in a specific pathogen-free environment (12/12 h light/dark cycle, 22°C–24°C, 40%–60% humidity) and fed freely. The mice were randomly divided into CON group, PAMK group, LPS group, and LPS + PAMK group. The other groups were gavaged with the same volume of saline to eliminate errors. Two weeks after gavage, the LPS group and LPS + PAMK group were injected intraperitoneally with LPS at a final concentration of 2 mg/kg, CON group and PAMK group were injected with normal saline to eliminate errors. Twelve hours post-LPS injection, all mice were euthanized using cervical dislocation, and liver tissues were rapidly collected. Portions of the liver tissue were fixed in 4% paraformaldehyde, with the remaining tissue immediately placed in liquid nitrogen and stored at −80°C for subsequent analysis.

### 2.4 H&E staining and Histopathological Evaluation of Liver Inflammation

The mouse liver samples were promptly immersed in a 4% solution of paraformaldehyde and allowed to remain undisturbed at ambient temperature for a duration of 7 days. Subsequently, the samples underwent a dehydration process prior to being encased in paraffin. From these paraffin blocks, thin sections of the tissue, each 4 μm in thickness, were carefully cut. These tissue sections were then subjected to hematoxylin and eosin staining. The stained slides were examined under an optical microscope to assess the tissue morphology. Two pathologists independently evaluated at least five randomly selected non-overlapping high-power fields (×400 magnification) using a modified Suzuki acute hepatitis scoring system (Chen et al., 2021; Suzuki et al., 1993). The scoring criteria comprised three parameters: (1) neutrophil infiltration (0–3 points) based on the number of neutrophils per field; (2) hepatocyte necrosis (0–3 points) ranging from spotty to confluent necrosis; and (3) inflammatory foci count (0–3 points) according to the number of discrete inflammatory clusters per field. The total inflammation score (0–9 points) represented the sum of these three components, with final scores calculated as the mean value across all evaluated fields. Any scoring discrepancies were resolved through joint re-evaluation and consensus between the pathologists.

### 2.5 Detection of serum biochemical indexes

The collected blood was incubated at 4°C for 1 h, then centrifuged at 3,000 rpm for 15 min. Afterwards, 100 μL of serum was collected and subjected to measurement of Aspartate aminotransferase (AST) and Alanine aminotransferase (ALT) using a biochemical analyzer.

### 2.6 Cell culture

RAW264.7 cells were purchased from the Chinese Typical Culture Collection Center (Wuhan, China). The cells were cultured in DMEM supplemented with 10% FBS and 1% penicillin/streptomycin at 37°C in 5% CO_2_. For experiments, cells were seeded at 5 × 10^4^ cells/well in 96-well plates (for CCK-8) or 1 × 10^6^ cells/well in 6-well plates (for qPCR/ELISA/WB) and allowed to adhere overnight (Monga S et al., 2022).

### 2.7 Cell counting Kit-8 (CCK-8) assay

Cell viability was assessed using the CCK-8 assay. Briefly, RAW264.7 cells were seeded in 96-well plates at a density of 5 × 10^3^ cells/well and incubated overnight. After treatment with different concentrations of PAMK (0, 1, 10, 20, 30, 40, 50, 100 μg/mL) for 24 h, 10 μL of CCK-8 reagent was added to each well and incubated for 2 h at 37°C. The optical density (OD) was measured at 450 nm using a microplate reader. Cell viability was calculated as follows:

$$\text{Cell viability }\left(\%\right)=\left({\text{OD}}_{\text{treatment}}-{\text{OD}}_{\text{blank}}\right)\;/\;\left({\text{OD}}_{\text{treatmen}}t-{\text{OD}}_{\text{blank}}\right)\times 100\%$$

### 2.8 Cell treatment

The RAW264.7 cells were divided into the following groups: CON group, LPS group (LPS was added to the culture medium to a final concentration of 20 ng/mL), PAMK group (PAMK was added to the culture medium to a final concentration of 20 μg/mL), LPS + PAMK group (LPS was added to the culture medium to a final concentration of 20 ng/mL, along with PAMK to a final concentration of 20 μg/mL, cells were pretreated with PAMK at 20 μg/mL for 24 h, followed by LPS addition to 20 ng/mL).

### 2.9 Plasmid construction and RNA oligonucleotide synthesis

The siRNA targeting GAS5 (si-GAS5; catalog number GZP22062100008, sequence 5′-GCC​UGG​AUG​GAG​GCU​CAA​ATT-3′) and a non-targeting control (si-NC) were both procured from Tsingke Biotechnology (Beijing, China). Additionally, the miR-223-3p mimic (sequence 5′-UGG​GGU​AUU​UGA​CAA​ACU​GAC​A-3′), its control (mimic-NC), the miR-223-3p inhibitor (sequence 5′-UGU​CAG​UUU​GUC​AAA​UAC​CCC​A-3′), and its corresponding control (inhibitor-NC) were crafted by RiboBio.

The GAS5 gene sequence was cloned into the pcDNA3.1 expression vector, which was purchased from Tsingke Biotechnology, utilizing the NheI and KpnI restriction enzyme sites. This vector is a widely used eukaryotic expression vector featuring a strong CMV promoter and multiple cloning sites for the insertion of genes of interest. Concurrently, a segment of GAS5 containing the miR-223-3p target site was inserted into the psiCHECK2 vector, also procured from Tsingke Biotechnology, at the SgfI/XhoI sites. The psiCHECK2 vector is a dual-luciferase reporter vector commonly used for the validation of miRNA targets.

Similarly, a 3′UTR segment of the NLRP3 gene that includes the miR-223-3p target sequence was integrated into the psiCHECK 2 vector at the SgfI/XhoI sites. A double fluorescence mutant of NLRP3 was also generated by mutating the miR-223-3p target site from ACUGAC to UGACAC.

### 2.10 Cell transfection

All transient transfections were performed using Lipofectamine^®^ 3,000 reagent according to the manufacturer’s instructions. The transfection doses of DNA in 12-well plates and 6-well plates were 1 μg/well and 2.5 μg/well, respectively. The final concentration of siRNA was 0.1 μM.

### 2.11 Dual luciferase reporter assay

The 293T cells (ATCC, Manassas, United States) were inoculated into 96-well culture plates. When the confluency degree reached 60%, miR-223-3p mimic or mimic NC was cotransfected into cells with NLRP3 and GAS5 wild-type and mutant double luciferase reporter vectors using Lipofectamine^®^ 3,000 reagent. After 48 h, the firefly luciferase and Renilla luminescence luciferase activities were detected by a dual luciferase reporter gene detection kit and a multifunctional enzyme standard.

### 2.12 ELISA

Collect the cell supernatant and use the IL-1β Mouse ELISA kit and IL-18 Mouse ELISA kit according to the manufacturer’s instructions to detect the levels of IL-1β and IL-18, respectively.

### 2.13 Quantitative real-time PCR

Total RNA was extracted from cells with TRIzol reagent. RNA was reverse transcribed to cDNA using the Reverse Transcription Kit following the manufacturer’s instructions. Subsequently, real-time PCR was performed using PowerUp™ SYBR™ Green Master Mix on the QuantStudio 7 Flex system (ABI, United States) to detect relative mRNA expression. The relative gene expression was calculated using the 2^−ΔΔCT^ method. Each experiment was repeated three times. The primers used in the study are listed in Supplementary Table S1. *GAPDH* was used as an internal reference for *GAS5*, *NLRP3*, *Caspase-1*, *IL-1*β, *IL-18* and *GSDMD*, and *U6* was used as an internal reference for *miR-223-3p*.

### 2.14 Western blot

RAW264.7 cells were inoculated into 6-well culture plates. When the confluency degree reached 60%, the overexpression vector, siRNA, miRNA, mimic and negative control were transfected into the cells. After 48 h, cell proteins were extracted using RIPA buffer and protease inhibitor mixture and then separated by 10% SDS-PAGE and immunoblotted using various antibodies according to standard Western blot procedures. Anti-NLRP3 (1:2000), anti-Caspase-1 (1:1000), anti-GSDMD (1:5000) and anti-GAPDH (1:5000) were incubated with protein blots at 4°C overnight. Subsequently, the secondary antibody (goat anti-rabbit) of 1:1000 dilution was use to incubate with the blots at 37°C for 1 h. Finally, chemiluminescence detection was carried out using the ECL Plus.

### 2.15 Detailed information of chemical reagents and kits

The chemical reagents and kits used in this study are listed in Supplementary Table S2.

### 2.16 Statistical analysis

Data were analyzed using SPSS 26.0 software, with one-way ANOVA followed by Tukey’s *post hoc* test for multiple comparisons or Student’s t-test for pairwise comparisons; quantitative data are expressed as mean ± SD, and significance was set at *P* < 0.05. In figures, ANOVA and t-test results are marked with different lowercase letters to indicate significant differences (*P* < 0.05). All graphs were generated using GraphPad Prism 7.0.

## 3 Results

### 3.1 Monosaccharide composition of PAMK

**FIGURE 1:**
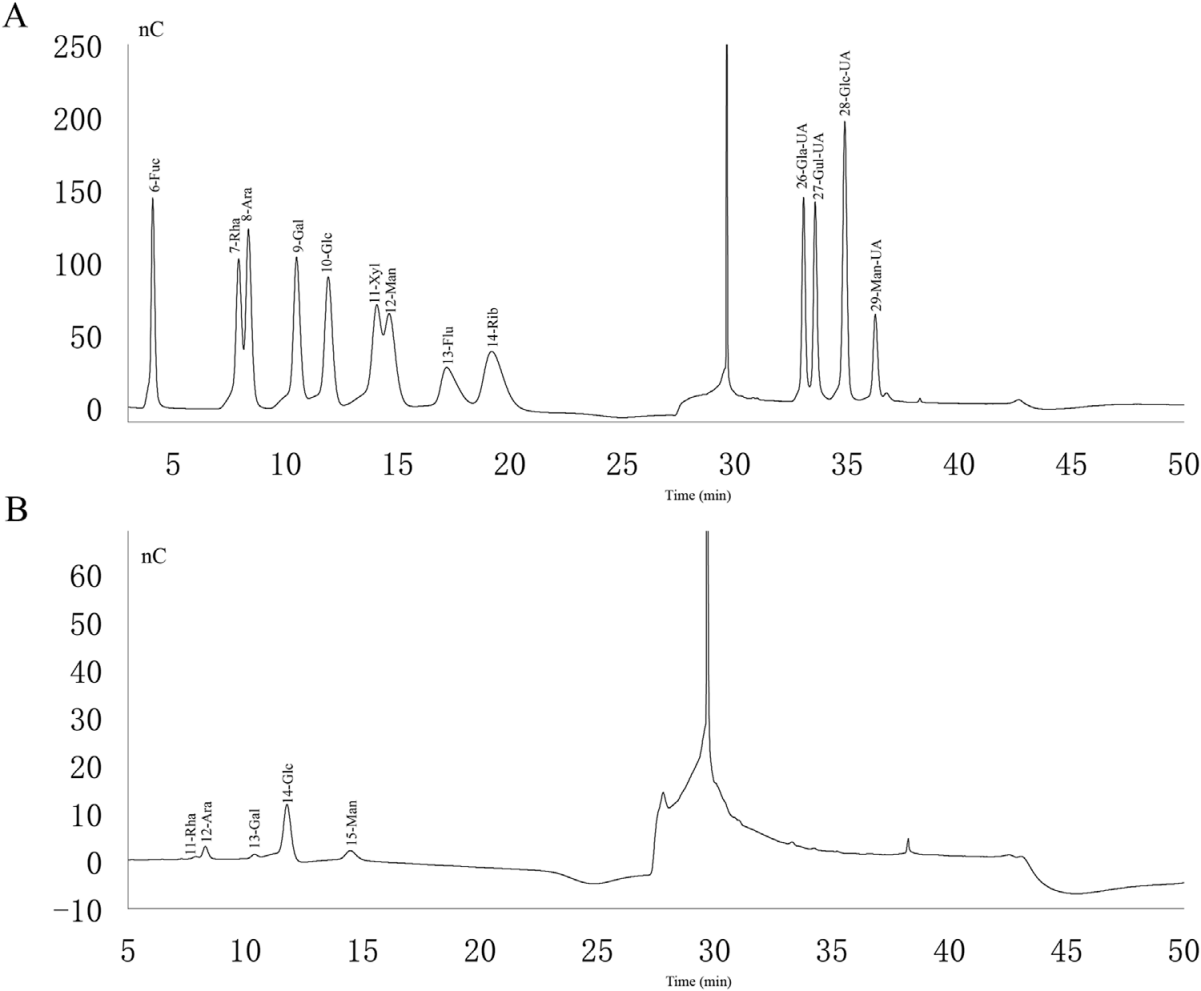
Ion chromatogram of standard and PAMK. **(A)** Ion chromatogram of standard. **(B)** Ion chromatogram of PAMK.

Analyzing the ion chromatograms of the standards and PAMK samples could confirmed that PAMK is mainly composed of Rha, Ara, Gal, Glc and Man (Figure 1), the proportions of which are 1.94%, 9.02%, 2.66%, 66.93% and 19.45%, Molar mass percentage of PAMK are 2.09%, 10.61%, 2.61%, 65.62% and 19.07%. (Table 1).

**TABLE 1:** Monosaccharide composition of PAMK.

Monosaccharide	Mass percentage	Molar mass percentage
Rha	1.94%	2.09%
Ara	9.02%	10.61%
Gal	2.66%	2.61%
Glc	66.93%	65.62%
Man	19.45%	19.07%

### 3.2 Histological observation of liver tissue in LPS-induced mice treated with PAMK

**FIGURE 2:**
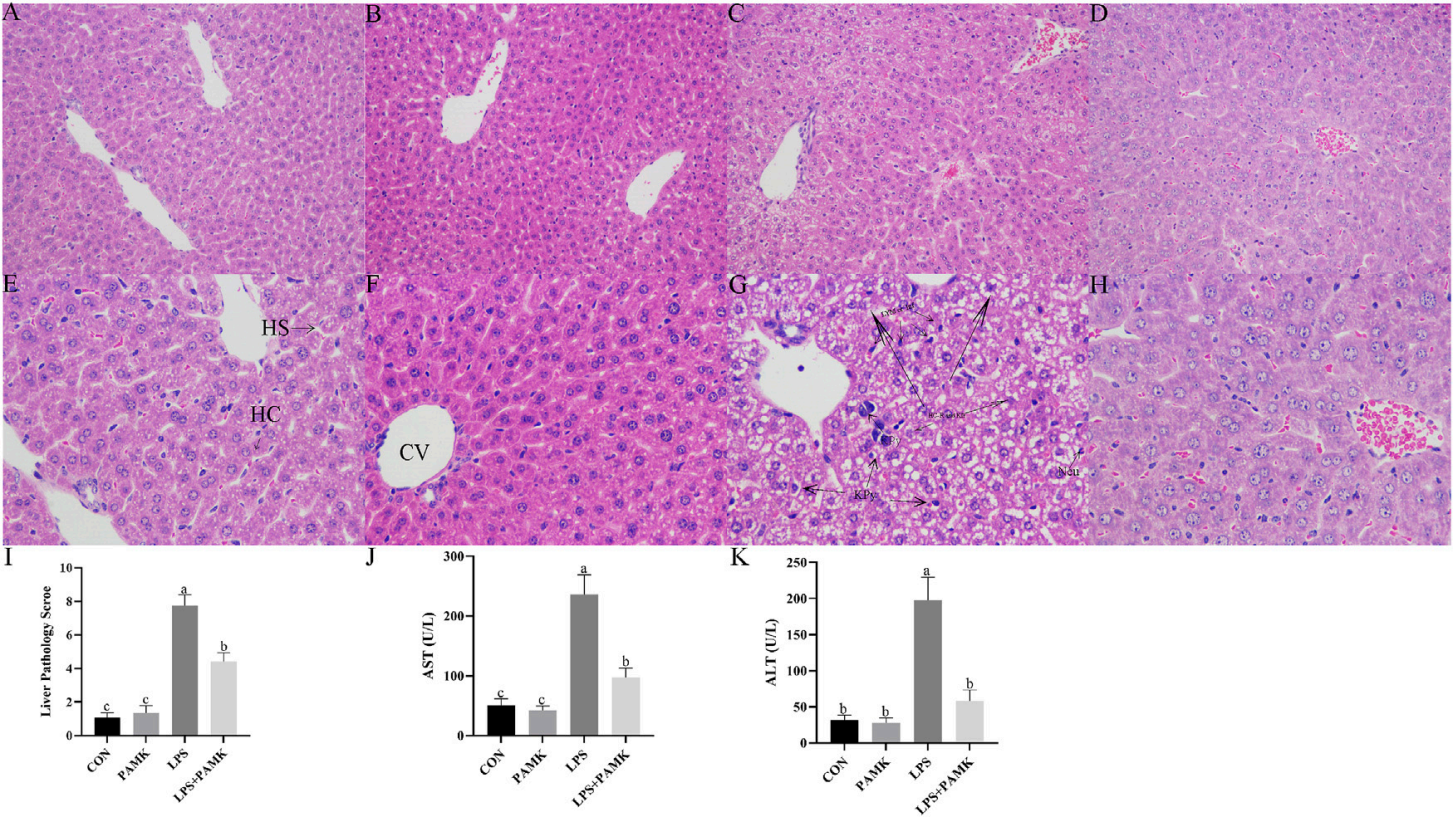
Histological observation of Mouse Liver Tissue Stained with H and E (**(A–D)** 200 ×, **(E–H)** 400 ×). CON Group: A, E; PAMK Group: B, F; LPS Group: C, G; LPS + PAMK Group: D, H. HS, Hepatic Sinusoid; HC, Hepatic Cord; CV, Central Vein; Triangle Symbol: Fat Droplets; KPy, Karyopyknosis; KIr, Nuclear irregularity; HC-R, Hepatocyte rupture; Neu, Neutrophils; Lym, Lymphocytes; Inf, Inflammatory infiltration; Nec, Necrotic area. **(I)** Histopathological Evaluation of Liver Inflammation; **(J)** AST; **(K)** ALT. Different letters indicate significant differences (*P* < 0.05).

Based on the results shown in Figure 2, the liver tissue of the CON and PAMK groups displayed well-organized and clear structures, with normal hepatocyte morphology. There was an abundance of binuclear hepatocytes, characterized by nearly round nuclei with visible nucleoli. In contrast, the liver tissue in the LPS group exhibited disrupted architecture, with evident loss of liver sinusoidal structure in some areas and noticeable expansion of the sinusoids. Abundant red blood cell aggregation was observed within the central veins and sinusoids, accompanied by a significant increase in lymphocyte numbers within the sinusoidal spaces. Infiltration of inflammatory cells, predominantly lymphocytes, surrounding the central veins was also evident. The primary pathological features included congestion, hemorrhage, and accumulation of inflammatory cells, specifically lymphocytes. Hepatocytes ruptured, nuclei densely stained and deformed with necrosis and apoptosis possible. In contrast, the liver tissue of the LPS + PAMK group demonstrated a restoration of near-normal structure, with clear visualization of the liver sinusoids, reduced extent of sinusoidal expansion, decreased hemorrhage and congestion, and absence of significant inflammatory cell accumulation. Notably, the hepatocytes exhibited a good recovery of morphological integrity, displaying normal nuclear shape without evidence of nuclear membrane rupture or hepatocyte necrosis. The histological results indicate that PAMK could effectively alleviate liver inflammatory damage caused by LPS in mice. The pathological score of liver tissues in the LPS group was markedly higher (*P* < 0.05) than that in other group; however, the liver pathology score in the LPS + PAMK group was lower than that in the LPS group (*P* < 0.05) (Figure 2I).

### 3.3 PAMK could regulate liver function abnormalities in mice induced by LPS

AST and ALT are two enzymes that reflect liver function. Increased levels of AST and ALT in serum represent the structure and function of the animal’s liver are damaged. In this study, according to Figures 2J,K, the serum AST and ALT level significantly increased in the LPS group compared to the CON group and PAMK group. However, after the addition of PAMK, the serum AST and ALT level significant decreased (*P* < 0.05).

### 3.4 The effect of PAMK on the expression of pyroptosis-associated genes and proteins, as well as cytokine levels in LPS-induced RAW264.7 cells

**FIGURE 3:**
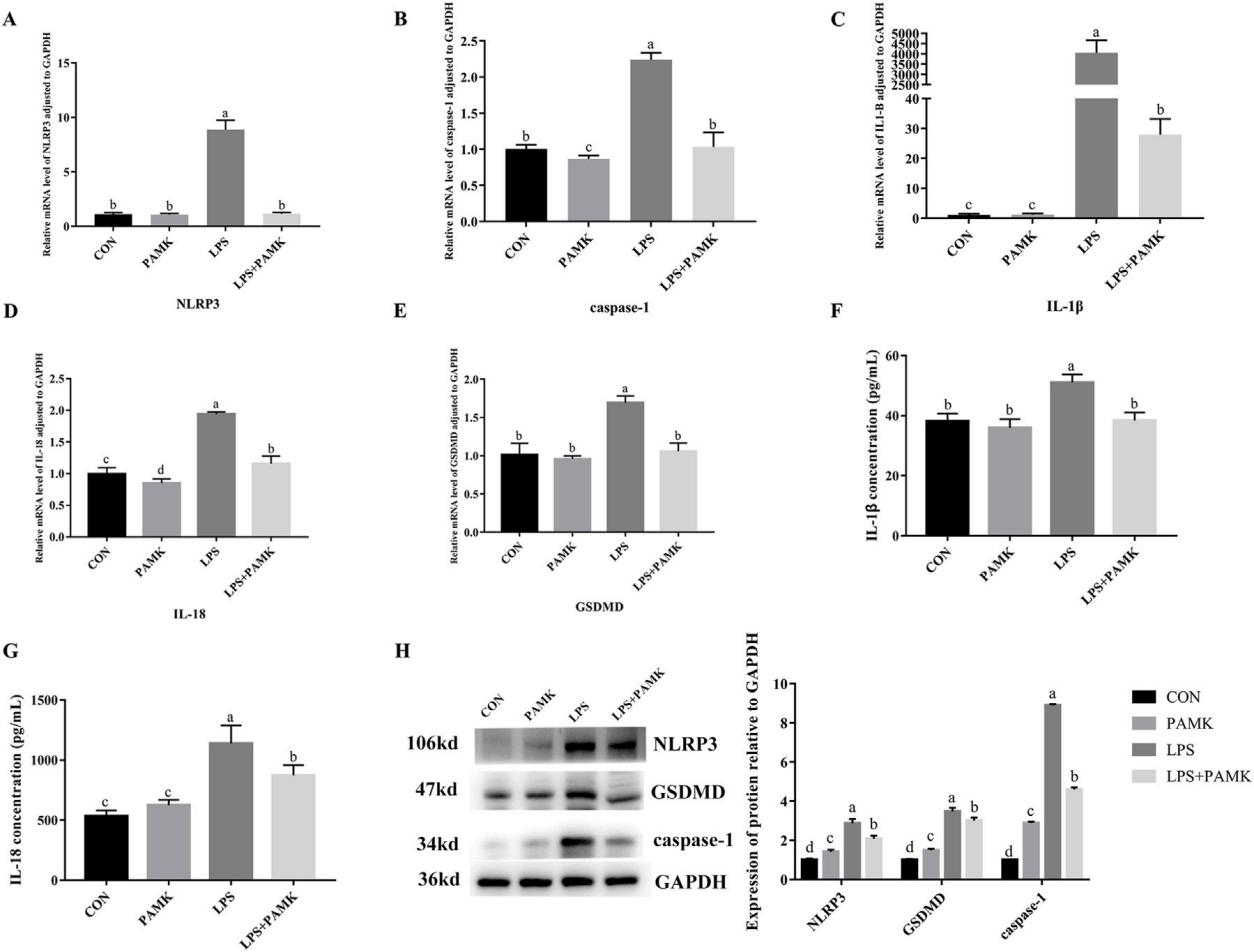
The effect of PAMK on the expression of pyroptosis-associated genes and proteins, as well as cytokine levels in LPS-induced raw264.7 cells. **(A)** Relative mRNA level of NLRP3 in cells from each experimental group after LPS treatment. **(B)** Relative mRNA level of caspase-1 in cells from each experimental group after LPS treatment. **(C)** Relative mRNA level of IL-1β in cells from each experimental group after LPS treatment. **(D)** Relative mRNA level of IL-18 in cells from each experimental group after LPS treatment. **(E)** Relative mRNA level of GSDMD in cells from each experimental group after LPS treatment. **(F)** The concentration of IL-1β in cell supernatant after LPS treatment. **(G)** The concentration of IL-18 in cell supernatant after LPS treatment. **(H)** Relative protein expression levels of cells in each group after LPS treatment. Different letters indicate significant differences (*P* < 0.05).

Macrophages are important inflammatory cells and play an important role in the development of inflammation. Therefore, we chose RAW264.7 cells to further explore the molecular mechanism of PAMK in alleviating LPS-induced liver inflammatory damage in mice. As shown in Figure 3, the mRNA expression levels of *NLRP3*, *caspase-1*, *IL-1*β, *IL-18*, and *GSDMD* were significantly increased in the LPS group compared to the CON group (*P* < 0.05). Similarly, the protein expression levels of NLRP3, caspase-1, and GSDMD were significantly elevated (*P* < 0.05). The levels of IL-1β and IL-18 cytokines in the cell supernatant were also markedly increased (*P* < 0.05). Conversely, in the LPS + PAMK group, the mRNA expression levels of *NLRP3*, *caspase-1*, *IL-18*, and *GSDMD* were significantly decreased compared to the LPS group (*P* < 0.05). The protein expression levels of NLRP3, caspase-1, and GSDMD were also significantly reduced (*P* < 0.05). Additionally, the levels of IL-1β and IL-18 cytokines in the cell supernatant were significantly decreased (*P* < 0.05). These findings indicated that PAMK could mitigate LPS-induced pyroptosis in RAW264.7 cells.

### 3.5 The effect of PAMK on miR-223-3p and GAS5 expression in LPS-induced RAW264.7 cells

**FIGURE 4:**
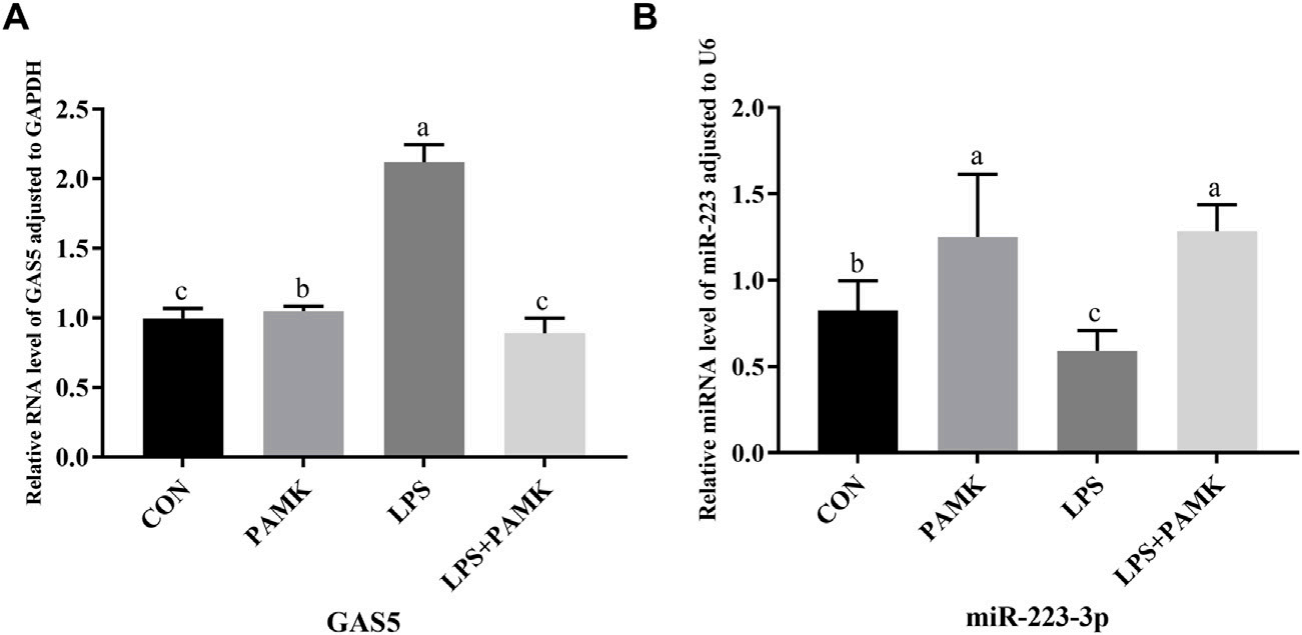
The effect of PAMK on miR-223-3p and GAS5 expression in LPS-induced RAW264.7 cells. **(A)** Relative RNA level of GAS5 in cells from each experimental group. **(B)** Relative miRNA level of miR-223-3p in cells from each experimental group. Different letters indicate significant differences (*P* < 0.05).

As shown in Figures 4A,B, the relative expression levels of *GAS5* were significantly increased in the LPS group compared to the CON group (*P* < 0.05), while the relative expression level of *miR-223-3p* significantly decreased (*P* < 0.05). Conversely, in the PAMK + LPS group, the relative expression level of *GAS5* significantly decreased compared to the LPS group (*P* < 0.05), while the relative expression level of *miR-223-3p* significantly increased (*P* < 0.05). These findings suggest that PAMK could modulate the expression of *GAS5* and *miR-223-3p* in RAW264.7 cells treated with LPS.

### 3.6 Investigation of targeted relationships among GAS5, miR-223-3p, and NLRP3

**FIGURE 5:**
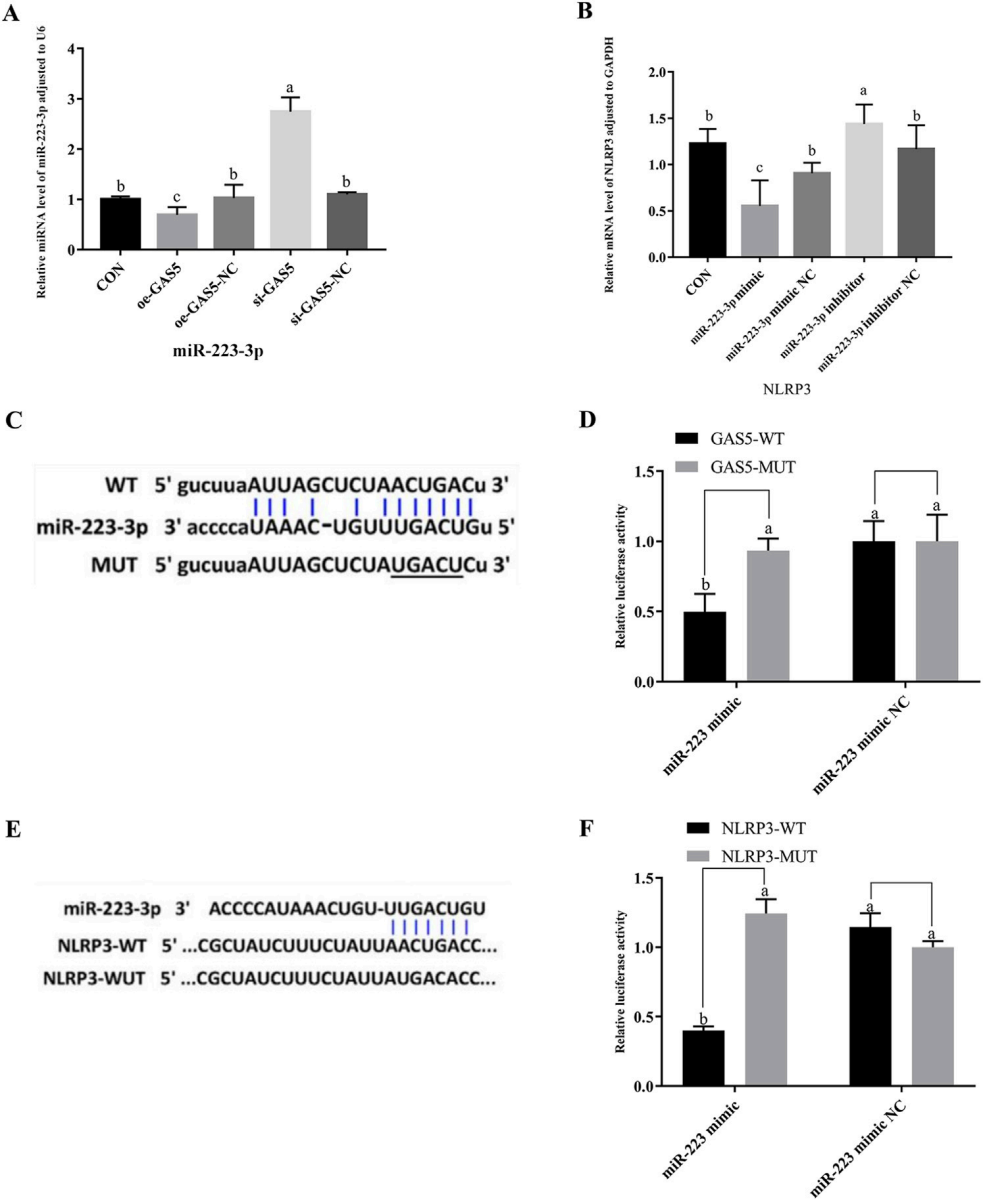
The relationship among GAS5, miR-223-3p, and NLRP3, and the shared targets between miR-223-3p and both GAS5 and NLRP3. **(A)** Relative miRNA level of miR-223-3p in cells from each experimental group. **(B)** Relative mRNA level of NLRP3 in cells from each experimental group. **(C)** and **(D)** The binding relationship between miR-223-3p and GAS5 was predicted by Starbase and further confirmed by dual-luciferase reporter assay. **(E)** and **(F)** The binding relationship between miR-223-3p and NLRP3 was predicted by Starbase and further confirmed by dual-luciferase reporter assay. Different letters indicate significant difference (*P* < 0.05).

As shown in Figures 5A,B, the expression of miR-223-3p significantly increased in the si-GAS5 group compared to the oe-GAS5 group (*P* < 0.05). Furthermore, the expression of *NLRP3* mRNA was significantly increased in the *miR-223-3p* inhibitor group compared to the *miR-223-3p* mimic group (*P* < 0.05).

Given the observed negative correlations between GAS5 and miR-223-3p, as well as between miR-223-3p and NLRP3 expression, we explored potential targeted interactions among these factors. Bioinformatics analysis revealed conserved binding sites between GAS5 and miR-223-3p (Figure 5C). Dual-luciferase reporter assays demonstrated that the miR-223-3p mimic significantly inhibited the luciferase activity of wild-type GAS5 (*P* < 0.05), but had no effect on mutant GAS5 (Figure 5D), confirming a direct targeting relationship between GAS5 and miR-223-3p. Furthermore, miR-223-3p was found to directly target the 3′UTR of NLRP3 mRNA (Figure 5E). Dual-luciferase reporter assays showed that the miR-223-3p mimic significantly inhibited the luciferase activity of wild-type NLRP3 (*P* < 0.05), but had no effect on mutant NLRP3, indicating a direct targeting relationship between miR-223-3p and NLRP3 (Figure 5F).

### 3.7 Effects of overexpression and inhibition of GAS5 on the transcription and protein expression of pyroptosis-related genes in RAW264.7 cells

**FIGURE 6:**
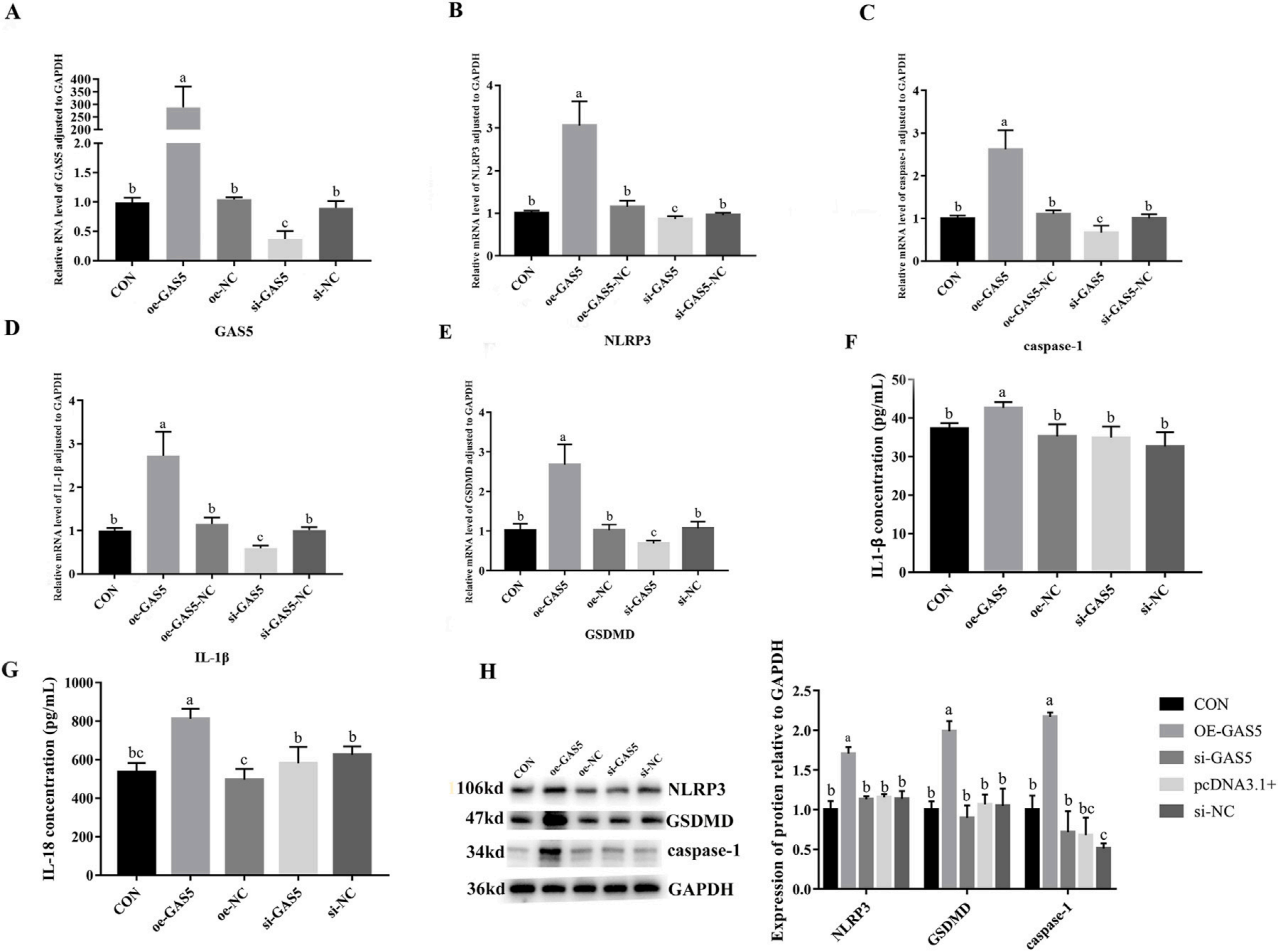
Effects of overexpression and inhibition of GAS5 on the transcription and protein expression of Pyroptosis-related genes in RAW264.7 cells. **(A)** Relative RNA level of GAS5 in cells in response to GAS5 genetic manipulation. **(B)** Relative mRNA level of NLRP3 in cells fin response to GAS5 genetic manipulation. **(C)** Relative mRNA level of caspase-1 in cells in response to GAS5 genetic manipulation. **(D)** Relative mRNA level of IL-1β in cells in response to GAS5 genetic manipulation. **(E)** Relative mRNA level of GSDMD in cells in response to GAS5 genetic manipulation. **(F)** The concentration of IL-1β in cell supernatant in response to GAS5 genetic manipulation. **(G)** The concentration of IL-18 in cell supernatant in response to GAS5 genetic manipulation. **(H)** Relative protein expression levels of cells in response to GAS5 genetic manipulation. Different letters indicate significant differences (*P* < 0.05).

To investigate the role of lncRNA GAS5 in cellular pyroptosis, we overexpressed (oe-GAS5) and inhibited (si-GAS5) *GAS5* in macrophages. As shown in Figure 6, the mRNA expression levels of *NLRP3*, *caspase-1*, *IL-1*β, *IL-18*, and *GSDMD* significantly increased in the oe-GAS5 group compared to the CON group (*P* < 0.05). Similarly, the protein expression levels of NLRP3, caspase-1, and GSDMD significantly increased in the oe-GAS5 group (*P* < 0.05). Additionally, the levels of IL-1β and IL-18 pro-inflammatory cytokines in the cell supernatant also increased. In contrast, the si-GAS5 group showed a significant decrease in the mRNA expression levels of NLRP3, caspase-1, IL-18, and GSDMD compared to the CON group (*P* < 0.05), as well as a significant reduction in the protein expression levels of NLRP3, caspase-1, and GSDMD (*P* < 0.05). These findings suggest that lncRNA GAS5 played a role in the pyroptosis process of RAW264.7 cells.

### 3.8 Effects of overexpression and inhibition of miR-223-3p on pyroptosis-related gene transcription and protein expression in RAW264.7 cells

**FIGURE 7:**
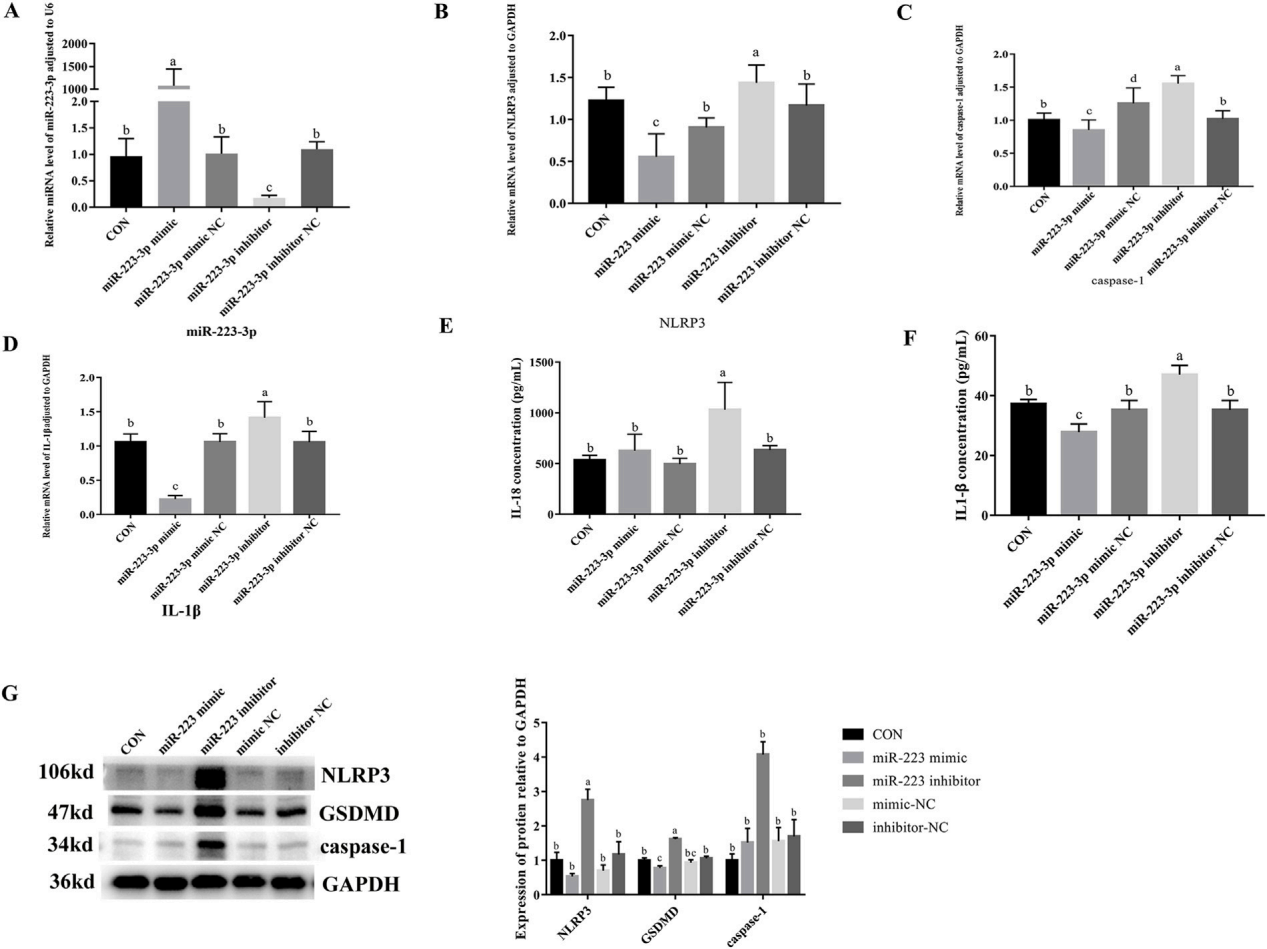
Effects of overexpression and inhibition of miR-223-3p on Pyroptosis-Related Gene transcription and protein expression in RAW264.7 Cells. **(A)** Relative miRNA level of miR-223-3p in cells in response to miR-223-3p genetic manipulation. **(B)** Relative mRNA level of NLRP3 in cells in response to miR-223-3p genetic manipulation. **(C)** Relative mRNA level of caspase-1 in cells in response to miR-223-3p genetic manipulation. **(D)** Relative mRNA level of IL-1β in cells in response to miR-223-3p genetic manipulation. **(E)** The concentration of IL-18 in cell supernatant in response to miR-223-3p genetic manipulation. **(F)** The concentration of IL-1β in cell supernatant in response to miR-223-3p genetic manipulation. **(G)** Relative protein expression levels of cells in response to miR-223-3p genetic manipulation. Different letters indicate significant differences (*P* < 0.05).

To investigate the role of *miR-223-3p* in cellular pyroptosis, we overexpressed (miR-223-3p mimic group) and inhibited (miR-223-3p inhibitor group) *miR-223-3p* in RAW264.7 cells. As shown in Figure 7, the mRNA expression levels of *NLRP3*, *caspase-1*, *IL-1*β, and *GSDMD* significantly decreased in the miR-223-3p mimic group compared to the CON group (*P* < 0.05). Similarly, the protein expression levels of NLRP3, caspase-1, and GSDMD significantly decreased in the miR-223-3p mimic group (*P* < 0.05). Conversely, in the miR-223-3p inhibitor group, the mRNA expression levels of *NLRP3*, *caspase-1*, *IL-1*β, and *GSDMD* significantly increased compared to the CON group (*P* < 0.05), along with a significant increase in the protein expression levels of NLRP3, caspase-1, and GSDMD (*P* < 0.05). These findings suggest that *miR-223-3p* played a crucial role in the regulation of pyroptosis in RAW264.7 cells.

### 3.9 Effects of GAS5 inhibition and miR-223-3p overexpression on pyroptosis-related gene transcription and expression in LPS-induced RAW264.7 cells

**FIGURE 8:**
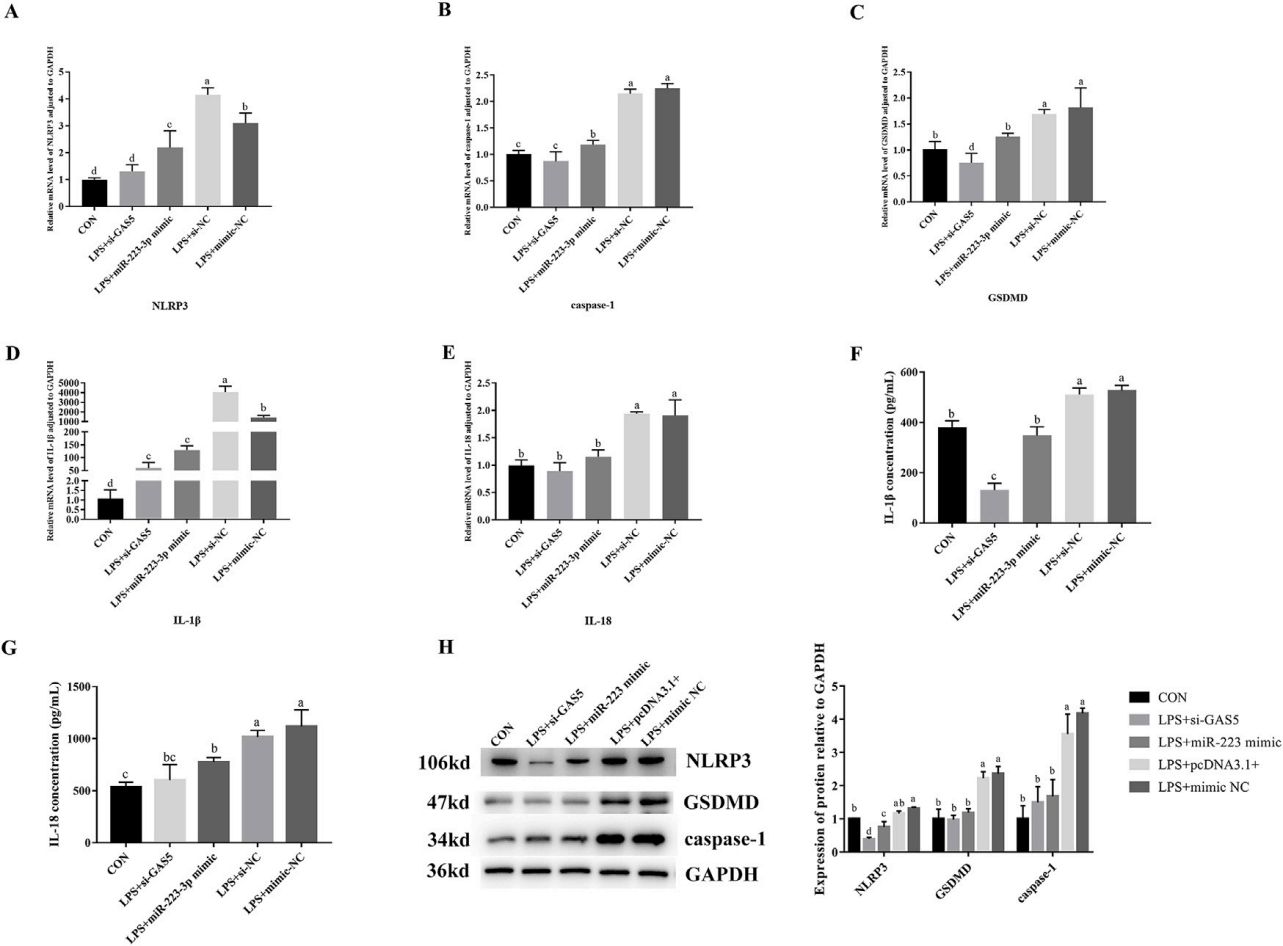
Effects of GAS5 inhibition and mir-223-3p overexpression on Pyroptosis-related gene transcription and expression in LPS-induced RAW264.7 cells. **(A–E)** Relative mRNA level of NLRP3, caspase-1, GSDMD, IL-1β, IL-18 in cells under combination treatments. **(F,G)** The concentration of IL-1β and IL-18 in cell supernatant under combination treatments. **(H)** Relative protein expression levels of cells under combination treatments. Different letters indicate significant differences (*P* < 0.05).

As shown in Figure 8, the downregulation of *GAS5* or the overexpression of *miR-223-3p* in LPS-treated RAW264.7 cells significantly decreased the relative mRNA expression levels of *NLRP3*, *caspase-1*, *IL-1*β, *IL-18*, and *GSDMD* if compared with the NC group (*P* < 0.05). Additionally, the protein expression levels of NLRP3, caspase-1, and GSDMD significantly reduced (*P* < 0.05), along with a significant decrease in the levels of pro-inflammatory cytokines IL-1β and IL-18 in the cell supernatant (*P* < 0.05). These findings indicated that both the inhibition of *GAS5* and the overexpression of *miR-223-3p* could suppress the pyroptosis process in RAW264.7 cells.

## 4 Discussion

The histomorphometric structure of the liver provides a visual assessment of liver health by identifying pathological changes such as inflammatory infiltration, steatosis, hepatic fibrosis, and hepatocellular necrosis (Wang et al., 2021). Studies have demonstrated that LPS can induce pathological changes in animal liver tissues. In mice induced with LPS, the liver exhibits significant inflammatory alterations, including congestion, disorganized hepatic cords, inflammatory cell infiltration, steatosis, cellular swelling, and vacuole-like changes (Guo et al., 2021; Miao et al., 2023). Consistent with these findings, our study also observed similar inflammatory changes in the liver. Liver sections from LPS-treated mice showed inflammatory cell aggregation around the central vein, characterized by congestion, hemorrhage, and accumulation of inflammatory cells, predominantly lymphocytes. However, the addition of PAMK resulted in notable improvements. In the PAMK group, hepatocytes maintained good morphology, and in the LPS + PAMK group, the liver tissue structure was nearly normal. The hepatic cords were clearly visible, the extent of sinusoidal expansion was reduced, and the levels of hemorrhage and congestion were decreased. No significant accumulation of inflammatory cells was observed. These results indicate that PAMK significantly alleviates LPS-induced inflammatory liver injury in mice.

When LPS enters the body, it recruits a large number of immune cells to the liver, stimulating these cells to secrete various pro-inflammatory cytokines, which exacerbate hepatocyte damage (Farghali et al., 2016). As inflammation progresses, it leads to hepatocyte death. Immune cells, particularly macrophages recruited to the liver, have been identified as key regulators of liver inflammation (Koyama and Brenner, 2017). These recruited macrophages are crucial components of liver inflammation and play vital roles in maintaining liver homeostasis and in the pathology of liver diseases (Rao et al., 2022). Damaged hepatocytes release damage-associated molecular patterns (DAMPs) to activate infiltrating macrophages. Activated macrophages then release various cytokines that directly damage hepatic parenchymal cells and enhance inflammatory cell infiltration (Rao et al., 2022). Targeting macrophage polarization and function is a promising therapeutic strategy, as regulating specific macrophage activities can help treat liver inflammation (Casari et al., 2023). Therefore, a deeper understanding of the mechanisms underlying macrophage-coordinated liver inflammation can provide valuable insights. In this experiment, we stimulated RAW264.7 cells with LPS. During this process, macrophages released large amounts of the inflammatory cytokines IL-1β and IL-18. Additionally, NLRP3, caspase-1, and GSDMD were activated in macrophages, which are typical features of pyroptosis.

Pyroptosis is a form of programmed cell death mediated by gasdermin family proteins. In the canonical pathway, caspase-1 is recruited and activated by the inflammasome, which then cleaves gasdermin D (GSDMD) to produce the active GSDMD-N terminal fragment. This fragment translocates to the cell membrane, forming pores that lead to pyroptosis (Dai et al., 2023). NLRP3 activation is implicated in various liver diseases. LPS stimulation can induce the activation of the NLRP3 inflammasome, leading to the maturation and release of inflammatory cytokines, thereby exacerbating liver injury (Seo et al., 2023; Sayaf et al., 2024). Studies have shown that once the NLRP3 inflammasome is activated by pathogenic microorganisms or endogenous danger signals, it promotes the maturation of pro-inflammatory cytokines such as IL-1β and IL-18 and secretes these mature cytokines outside the cell (Kelley et al., 2019). Subsequently, a broader range of inflammatory cytokines, including IL-6, IL-10, and TNF-α, are activated, resulting in an excessive inflammatory response (Gao et al., 2019). Research indicates that LPS can induce pyroptosis in bone marrow-derived macrophages by upregulating NLRP3, caspase-1, and GSDMD, leading to the release of inflammatory cytokines (Hu et al., 2024). Similarly, in LPS-treated RAW264.7 cells, the upregulation of NLRP3, caspase-1, and GSDMD, along with the maturation and secretion of IL-1β and IL-18, further confirms the occurrence of pyroptosis (Sun et al., 2023). In this study, we obtained similar conclusions: LPS induced a significant upregulation of the NLRP3, caspase-1, and GSDMD at both the gene and protein levels in cells, accompanied by a marked increase in the levels of the inflammatory cytokines IL-18 and IL-1β. These findings suggest that LPS triggers pyroptosis in RAW264.7 cells.

Macrophages play a critical role in innate immunity, with approximately 90% of the total macrophage population in the human body residing in the liver. Studies have shown that macrophage pyroptosis can cause damage to internal organs. When pyroptosis is activated in macrophages, atherosclerosis can be induced in mice (Zeng et al., 2021). Wei further confirmed that there is an important connection between macrophage pyroptosis and atherosclerosis (Wei et al., 2023). Silica can also induce pyroptosis in macrophages of lung and cause lung inflammation in mice (Yin et al., 2022). Macrophage pyroptosis also plays an important role in the acute injury of mouse liver, and inhibiting macrophage pyroptosis can effectively alleviate liver damage (Chen R X et al., 2023). In our research, PAMK was found to reduce the expression levels of NLRP3, caspase-1, and GSDMD in LPS-induced macrophages, along with a decrease in the levels of the inflammatory cytokines IL-1β and IL-18. Since pyroptosis is often accompanied by the release of large amounts of inflammatory cytokines, these results suggest that PAMK might alleviate the excessive release of inflammatory cytokines by reducing macrophage pyroptosis. This reduction in inflammatory cytokine release could lead to decreased liver inflammatory.

GAS5, a long non-coding RNA encoded by the GAS5 gene, has recently been identified as a tumor suppressor in several types of cancer (Xu et al., 2016). GAS5 also plays a role in inflammation and NLRP3-induced programmed cell necrosis. Studies have shown that overexpression of the lncRNA GAS5 in LPS-stimulated cardiac fibrosis models and pyroptosis models of cardiac fibroblasts can increase and suppress the programmed necrosis of these cells, respectively. Additionally, it has been shown to reduce the expression of caspase-1 and NLRP3 in cardiac fibroblasts, demonstrating that the methylation of DNMT1 by GAS5 can lead to programmed necrosis of cardiac fibroblasts by affecting the NLRP3 axis (She et al., 2020). In this study, we similarly found that GAS5 significantly upregulated in macrophages stimulated by LPS. Further investigation revealed that GAS5 can activate the pyroptosis process, increasing the expression levels of pyroptosis-related factors and exacerbating the inflammatory response in macrophages. Conversely, PAMK was able to downregulate GAS5 and reduce the expression levels of pyroptosis-related factors. These results strongly suggest that GAS5 is involved in the process of pyroptosis and inflammation, and that PAMK can alleviate pyroptosis by regulating GAS5, thereby reducing inflammation.

MiR-223, another significant non-coding RNA within the microRNAs family, has also been reported to play a crucial role in disease pathology. In recent studies, miR-223-3p has been identified to ameliorate experimental autoimmune myocarditis and inflammatory damage in retinal pigment epithelium by targeting NLRP3 (Hu et al., 2019; Chen et al., 2020). Similarly, in liver tissue, miR-223-3p has been confirmed to target NLRP3, thereby alleviating LPS-mediated inflammatory liver injury in goslings, where LPS stimulation leads to a decrease in the expression of miR-223-3p (Chen F et al., 2023). In this study, our results also showed that miR-223-3p was significantly downregulated in LPS-stimulated RAW264.7 cells. Additionally, miR-223-3p targets NLRP3, and its overexpression significantly inhibited the activation of the NLRP3 inflammasome and the expression of caspase-1, IL-1β, IL-18, and GSDMD, indicating that miR-223-3p plays a role in the pyroptosis process. Furthermore, PAMK can inhibit pyroptosis by regulating miR-223-3p, thereby alleviating inflammation.

An increasing body of research indicates that lncRNAs can sponge their target miRNAs, thereby negatively regulating the expression of miRNAs (Tay et al., 2014). This mechanism is also applicable to inflammation and has garnered widespread attention. Recent studies have found that GAS5 may act as a competing endogenous RNA (ceRNA) for miR-223. Knockdown of GAS5 can partially reverse the effects of miR-223 inhibitors on cell proliferation, cell cycle distribution, and programmed cell necrosis (Yao et al., 2019; Dong et al., 2018). In this study, we identified binding sites for miR-223-3p with both NLRP3 and GAS5, and validated these targeting relationships using dual-luciferase reporter assays, which align with recent research. Additionally, inhibition of GAS5 significantly increased miR-223-3p expression, inhibited the upregulation of inflammatory cytokines, and suppressed the activation of the NLRP3 inflammasome. This indicates that the GAS5/miR-223-3p axis is vital in regulating the progression of pyroptosis. Furthermore, our findings revealed that miR-223-3p negatively regulates NLRP3, thereby controlling the expression of downstream inflammatory cytokines. This demonstrates that GAS5 can regulate NLRP3 and its downstream factors IL-1β and IL-18 by targeting miR-223-3p, uncovering a pathway involving GAS5/miR-223-3p/NLRP3 in the process of pyroptosis.

## 5 Conclusion

**FIGURE 9:**
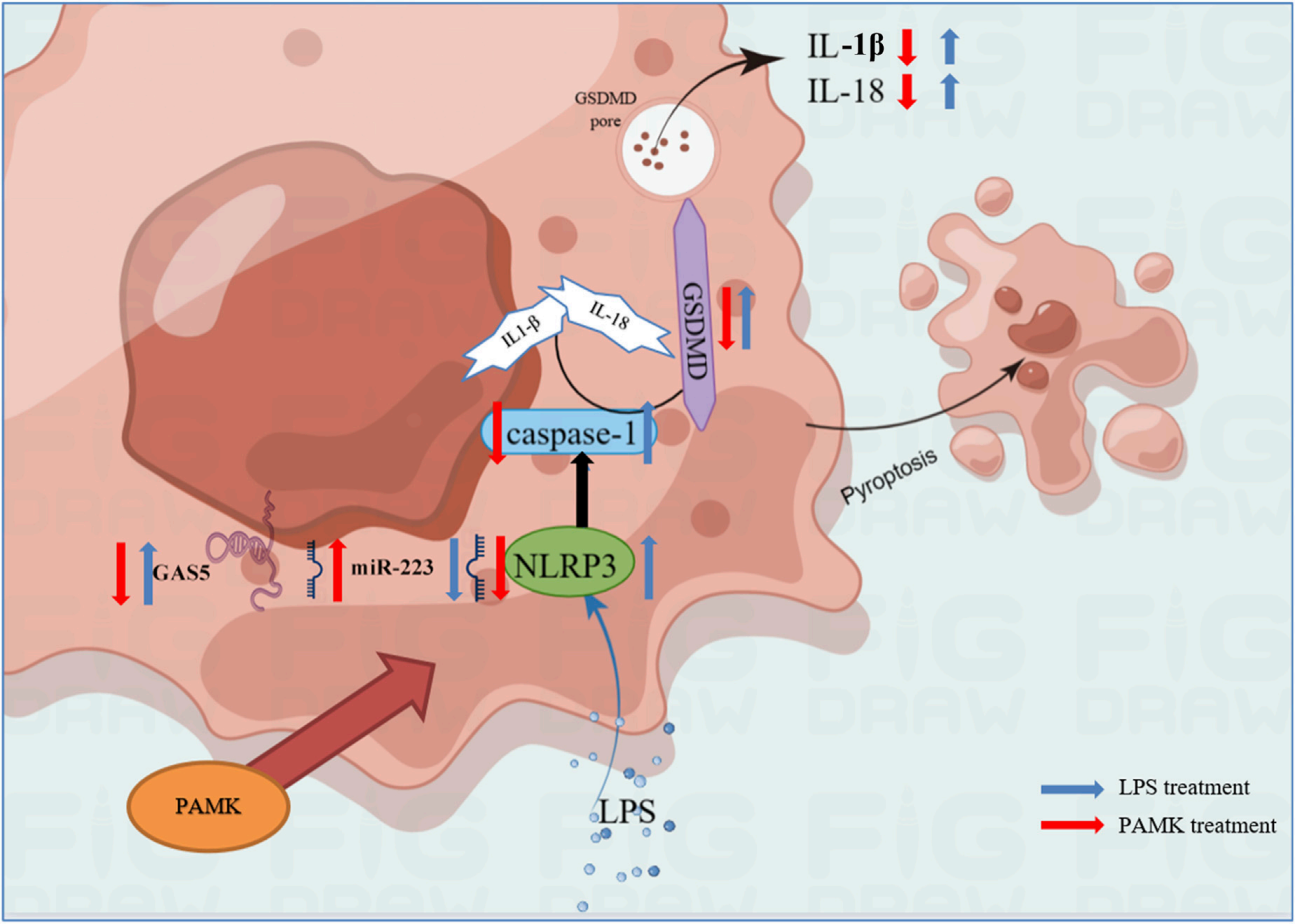
Schematic representation of the mechanism by which PAMK regulates NLRP3 via the lncRNA GAS5/miR-223 axis to alleviate LPS-induced pyroptosis in RAW264.7 cells.

In summary, the results of this study indicate that PAMK can alleviate LPS-induced disruption of normal liver physiological structure and reduce liver inflammatory injury. GAS5 is upregulated in LPS-stimulated macrophages and promotes the expression of pyroptosis-related genes and the release of inflammatory cytokines by targeting the miR-223-3p/NLRP3 axis. *In vitro* inhibition of GAS5 significantly suppresses the activation of pyroptosis-related genes and proteins, as well as the activation and release of inflammatory cytokines. PAMK inhibits GAS5 while upregulating miR-223-3p, significantly suppressing NLRP3 activation and the expression of pyroptosis-related proteins and genes, and reducing the release of inflammatory cytokines. These findings provide theoretical evidence that PAMK can inhibit macrophage pyroptosis and thereby mitigate liver inflammation (Figure 9).

## Data Availability

The raw data supporting the conclusions of this article will be made available by the authors, without undue reservation.

## References

[B1] Bauernfeind F. Rieger A. Schildberg F. A. Knolle P. A. Schmid-Burgk J. L. Hornung V. (2012). NLRP3 inflammasome activity is negatively controlled by miR-223. J. Immunol. 189 (8), 4175–4181. 10.4049/jimmunol.1201516 22984082

[B2] Boaru S. G. Borkham-Kamphorst E. Tihaa L. Haas U. Weiskirchen R. (2012). Expression analysis of inflammasomes in experimental models of inflammatory and fibrotic liver disease. J. Inflamm.-Lond. 9 (1), 49. 10.1186/1476-9255-9-49 23192004 PMC3599703

[B3] Broz P. Pelegrin P. Shao F. (2020). The gasdermins, a protein family executing cell death and inflammation. Nat. Rev. Immunol. 20 (3), 143–157. 10.1038/s41577-019-0228-2 31690840

[B4] Casari M. Siegl D. Deppermann C. Schuppan D. (2023). Macrophages and platelets in liver fibrosis and hepatocellular carcinoma. Front. Immunol. 14, 1277808. 10.3389/fimmu.2023.1277808 38116017 PMC10728659

[B5] Chen L. Hou X. Zhang M. Zheng Y. Zheng X. Yang Q. (2020). MicroRNA-223-3p modulates dendritic cell function and ameliorates experimental autoimmune myocarditis by targeting the NLRP3 inflammasome. Mol. Immunol. 117, 73–83. 10.1016/j.molimm.2019.10.027 31743855

[B6] Chen R. X. Jiang W. J. Liu S. C. Wang Z. Y. Wang Z. B. Zhou T. (2023). Apolipoprotein A-1 protected hepatic ischaemia-reperfusion injury through suppressing macrophage pyroptosis via TLR4-NF-κB pathway. Liver Int. 43 (1), 234–248. 10.1111/liv.15448 36203339

[B7] Chen S. N. Tan Y. Xiao X. C. Li Q. Wu Q. Peng Y. Y. (2021). Deletion of TLR4 attenuates lipopolysaccharide-induced acute liver injury by inhibiting inflammation and apoptosis. Acta Pharmacol. Sin. 42 (10), 1610–1619. 10.1038/s41401-020-00597-x 33495514 PMC8463538

[B8] Chen F. Li B. Li W. Chen W. Huang Y. Tian Y. (2023). Polysaccharide of Atractylodes macrocephala koidz alleviate lipopolysaccharide-stimulated liver inflammation injury of goslings through miR-223/NLRP3 axis. Poult. Sci. 102 (1), 102285. 10.1016/j.psj.2022.102285 36436369 PMC9706645

[B9] Cho S. J. Hong K. S. Jeong J. H. Lee M. Choi A. Stout-Delgado H. W. (2019). DROSHA-dependent AIM2 inflammasome activation contributes to lung inflammation during idiopathic pulmonary fibrosis. Cells 8 (8), 938. 10.3390/cells8080938 31434287 PMC6721825

[B10] Dai Z. Liu W. C. Chen X. Y. Wang X. Li J. L. Zhang X. (2023). Gasdermin D-mediated pyroptosis: mechanisms, diseases, and inhibitors. Front. Immunol. 14, 1178662. 10.3389/fimmu.2023.1178662 37275856 PMC10232970

[B11] Dong X. Kong C. Liu X. Bi J. Li Z. Li Z. (2018). GAS5 functions as a ceRNA to regulate hZIP1 expression by sponging miR-223 in clear cell renal cell carcinoma. Am. J. Cancer Res. 8 (8), 1414–1426. 30210913 PMC6129482

[B12] Farghali H. Kgalalelo K. M. Wojnarova L. Kutinova C. N. (2016). *In vitro* and *in vivo* experimental hepatotoxic models in liver research: applications to the assessment of potential hepatoprotective drugs. Physiol. Res. 65 (Suppl. 4), S417–S425. 10.33549/physiolres.933506 28006924

[B13] Gao Y. Wu X. Zhao S. Zhang Y. Ma H. Yang Z. (2019). Melatonin receptor depletion suppressed hCG-induced testosterone expression in mouse leydig cells. Cell. Mol. Biol. Lett. 24 (1), 21. 10.1186/s11658-019-0147-z 30915128 PMC6416941

[B14] Guo S. Li W. Chen F. Yang S. Huang Y. Tian Y. (2021). Polysaccharide of Atractylodes macrocephala koidz regulates LPS-mediated mouse hepatitis through the TLR4-MyD88-NFκB signaling pathway. Int. Immunopharmacol. 98, 107692. 10.1016/j.intimp.2021.107692 34116287

[B15] Hu X. Zou M. Zheng W. Zhu M. Hou Q. Gao H. (2024). Bhlhe40 deficiency attenuates LPS-induced acute lung injury through preventing macrophage pyroptosis. Respir. Res. 25 (1), 100. 10.1186/s12931-024-02740-2 38402153 PMC10894472

[B16] Hu Z. Lv X. Chen L. Gu X. Qian H. Fransisca S. (2019). Protective effects of microRNA-22-3p against retinal pigment epithelial inflammatory damage by targeting NLRP3 inflammasome. J. Cell. Physiol. 234 (10), 18849–18857. 10.1002/jcp.28523 30927257

[B17] Huppertz C. Jager B. Wieczorek G. Engelhard P. Oliver S. J. Bauernfeind F. G. (2020). The NLRP3 inflammasome pathway is activated in sarcoidosis and involved in granuloma formation. Eur. Respir. J. 55 (3), 1900119. 10.1183/13993003.00119-2019 31949113

[B18] Kelley N. Jeltema D. Duan Y. He Y. (2019). The NLRP3 inflammasome: an overview of mechanisms of activation and regulation. Int. J. Mol. Sci. 20 (13), 3328. 10.3390/ijms20133328 31284572 PMC6651423

[B19] Koyama Y. Brenner D. A. (2017). Liver inflammation and fibrosis. J. Clin. Invest. 127 (1), 55–64. 10.1172/JCI88881 28045404 PMC5199698

[B20] Miao F. Geng S. Ning D. (2023). Hydroxytyrosol ameliorates LPS-induced acute liver injury (ALI) in mice by modulating the balance between M1/M2 phenotype macrophage and inhibiting TLR4/NF-κB activation. J. Funct. Foods. 102, 105455. 10.1016/j.jff.2023.105455

[B21] Mo R. Li J. Chen Y. Ding Y. (2022). lncRNA GAS5 promotes pyroptosis in COPD by functioning as a ceRNA to regulate the miR-223-3p/NLRP3 axis. Mol. Med. Rep. 26 (1), 219. 10.3892/mmr.2022.12735 35583006 PMC9175270

[B22] Monga S. Fares B. Yashaev R. Melamed D. Kahana M. Fares F. (2022). The effect of natural-based formulation (NBF) on the response of RAW264.7 macrophages to LPS as an *in vitro* model of inflammation. J. Fungi (Basel) 8 (3), 321. 10.3390/jof8030321 35330323 PMC8955716

[B23] Rao J. Wang H. Ni M. Wang Z. Wang Z. Wei S. (2022). FSTL1 promotes liver fibrosis by reprogramming macrophage function through modulating the intracellular function of PKM2. Gut 71 (12), 2539–2550. 10.1136/gutjnl-2021-325150 35140065 PMC9664121

[B24] Sayaf K. Battistella S. Russo F. P. (2024). NLRP3 inflammasome in acute and chronic liver diseases. Int. J. Mol. Sci. 25 (8), 4537. 10.3390/ijms25084537 38674122 PMC11049922

[B25] Seo H. Y. Lee S. H. Park J. Y. Han E. Han S. Hwang J. S. (2023). Lobeglitazone inhibits LPS-induced NLRP3 inflammasome activation and inflammation in the liver. PLoS One 18 (8), e0290532. 10.1371/journal.pone.0290532 37616215 PMC10449201

[B26] She Q. Shi P. Xu S. S. Xuan H. Y. Tao H. Shi K. H. (2020). DNMT1 methylation of LncRNA GAS5 leads to cardiac fibroblast pyroptosis via affecting NLRP3 axis. Inflammation 43 (3), 1065–1076. 10.1007/s10753-020-01191-3 32008164

[B27] Shi X. Xie X. Sun Y. He H. Huang H. Liu Y. (2020). Paeonol inhibits NLRP3 mediated inflammation in rat endothelial cells by elevating hyperlipidemic rats plasma exosomal miRNA-223. Eur. J. Pharmacol. 885, 173473. 10.1016/j.ejphar.2020.173473 32800809

[B28] Sun K. Li Y. Y. Jin J. (2021). A double-edged sword of immuno-microenvironment in cardiac homeostasis and injury repair. Signal Transduct. Target. Ther. 6 (1), 79. 10.1038/s41392-020-00455-6 33612829 PMC7897720

[B29] Sun S. Gong D. Liu R. Wang R. Chen D. Yuan T. (2023). Puerarin inhibits NLRP3-Caspase-1-GSDMD-Mediated pyroptosis via P2X7 receptor in cardiomyocytes and macrophages. Int. J. Mol. Sci. 24 (17), 13169. 10.3390/ijms241713169 37685976 PMC10488171

[B30] Suzuki S. Toledo-Pereyra L. H. Rodriguez F. J. Cejalvo D. (1993). Neutrophil infiltration as an important factor in liver ischemia and reperfusion injury. Modulating effects of FK506 and cyclosporine. Transplantation 55 (6), 1265–1272. 10.1097/00007890-199306000-00011 7685932

[B31] Tay Y. Rinn J. Pandolfi P. P. (2014). The multilayered complexity of ceRNA crosstalk and competition. Nature 505 (7483), 344–352. 10.1038/nature12986 24429633 PMC4113481

[B32] Wang Y. Brodin E. Nishii K. Frieboes H. B. Mumenthaler S. M. Sparks J. L. (2021). Impact of tumor-parenchyma biomechanics on liver metastatic progression: a multi-model approach. Sci. Rep. 11 (1), 1710. 10.1038/s41598-020-78780-7 33462259 PMC7813881

[B33] Wei Y. Lan B. Zheng T. Yang L. Zhang X. Cheng L. (2023). GSDME-mediated pyroptosis promotes the progression and associated inflammation of atherosclerosis. Nat. Commun. 14 (1), 929. 10.1038/s41467-023-36614-w 36807553 PMC9938904

[B34] Wu Q. Li B. Li Y. Liu F. Yang L. Ma Y. (2022). Effects of PAMK on lncRNA, miRNA, and mRNA expression profiles of thymic epithelial cells. Genomics 22 (5), 849–863. 10.1007/s10142-022-00863-7 35505120

[B35] Xu C. Zhang Y. Wang Q. Xu Z. Jiang J. Gao Y. (2016). Long non-coding RNA GAS5 controls human embryonic stem cell self-renewal by maintaining NODAL signalling. Nat. Commun. 7, 13287. 10.1038/ncomms13287 27811843 PMC5097163

[B36] Xu W. Zhang L. Geng Y. Liu Y. Zhang N. (2020). Long noncoding RNA GAS5 promotes microglial inflammatory response in parkinson's disease by regulating NLRP3 pathway through sponging miR-223-3p. Int. Immunopharmacol. 85, 106614. 10.1016/j.intimp.2020.106614 32470877

[B37] Yao J. Shi Z. Ma X. Xu D. Ming G. (2019). lncRNA GAS5/miR-223/NAMPT axis modulates the cell proliferation and senescence of endothelial progenitor cells through PI3K/AKT signaling. J. Cell. Biochem. 120 (9), 14518–14530. 10.1002/jcb.28713 31026096

[B38] Yin H. Fang L. Wang L. Xia Y. Tian J. Ma L. (2022). Acute silica exposure triggers pulmonary inflammation through macrophage pyroptosis: an experimental simulation. Front. Immunol. 13, 874459. 10.3389/fimmu.2022.874459 35464414 PMC9021383

[B39] Zeng W. Wu D. Sun Y. Suo Y. Yu Q. Zeng M. (2021). The selective NLRP3 inhibitor MCC950 hinders atherosclerosis development by attenuating inflammation and pyroptosis in macrophages. Sci. Rep. 11 (1), 19305. 10.1038/s41598-021-98437-3 34588488 PMC8481539

[B40] Zhao G. Jiang K. Yang Y. Zhang T. Wu H. Shaukat A. (2018). The potential therapeutic role of miR-223 in bovine endometritis by targeting the NLRP3 inflammasome. Front. Immunol. 9, 1916. 10.3389/fimmu.2018.01916 30186287 PMC6113393

