# Supplementary material for: Polysaccharide of Atractylodes macrocephala Koidz alleviate LPS-induced inflammatory liver injury by reducing pyroptosis of macrophage via regulating LncRNA GAS5/miR-223-3p/NLRP3 axis
Source: Front Pharmacol. 2025 Jul 29;16:1593689. doi: 10.3389/fphar.2025.1593689 (PMC12339467; doi:10.3389/fphar.2025.1593689)
Supplement: Supplementary file 1 [file Table1.docx]

Table S1 Primer information

| Genes | Primer sequences (5'-3') | Accession number |
| --- | --- | --- |
| *GAPDH* | F: GGGGACCAAGGAGTTTAAA  R: GCGGGTCCTGTTAATTTGG | NM_001289726.2 |
| *IL-1β* | F: GCAACTGTTCCTGAACTCAACT  R: ATCTTTTGGGGTCCGTCAACT | NM_008361.4 |
| *IL-18* | F: AACTGCAGACTGGCACAGC  R: AGGTTTGAGGCGGCTTTCTT | XM_036154618.1 |
| *NLRP3* | F: CAAGGCTGCTATCTGGAGGAA  R: TGCAACGGACACTCGTCATC | NM_001359638.1 |
| *Caspase-1* | F: CCAGGAGGGAATATGTGGGAC  R: ACTCCTTGTTTCTCTCCACGG | NM_009807.2 |
| *GSDMD* | F: AGTGCTCCAGAACCAGAACCG  R: TCTCCCATGCCTGACAACATC | NM_026960.4 |
