# Supplementary material for: Polysaccharide of Atractylodes macrocephala Koidz alleviate LPS-induced inflammatory liver injury by reducing pyroptosis of macrophage via regulating LncRNA GAS5/miR-223-3p/NLRP3 axis
Source: Front Pharmacol. 2025 Jul 29;16:1593689. doi: 10.3389/fphar.2025.1593689 (PMC12339467; doi:10.3389/fphar.2025.1593689)
Supplement: Supplementary file 2 [file Table2.docx]

Table S2 Specifications of experimental materials

| Product Name | Catalog Number | Supplier | Origin |
| --- | --- | --- | --- |
| Lipopolysaccharide (LPS) | L2880 | Sigma-Aldrich | USA |
| PAMK | CY201216 | Xi'an Tianyuan BioProducts | China |
| DMEM | C11995500BT | Gibco | USA |
| Fetal Bovine Serum (FBS) | 10100147 | Gibco | USA |
| Penicillin/Streptomycin | 151400122 | Gibco | USA |
| Lipofectamine®3000 | L3000015 | Invitrogen | USA |
| IL-1β Mouse ELISA Kit | E-EL-M0037c | Elabscience | China |
| IL-18 Mouse ELISA Kit | E-EL-M0730c | Elabscience | China |
| RIPA buffer | P0013B | Beyotime | China |
| Protease inhibitor | P1005 | Beyotime | China |
| Anti-NLRP3 | WL2635 | Wanleibio | China |
| Anti-Caspase-1 | 22915-1-AP | Proteintech | China |
| Anti-GSDMD | 66387-1-lg | Proteintech | China |
| Anti-GAPDH | 109494-1-AP | Proteintech | China |
| HRP-goat anti-rabbit | A0208 | Beyotime | China |
| ECL Plus | P0018S | Beyotime | China |
| TRIzol Reagent | 15596-026 | Invitrogen | USA |
| SYBR™ Green Master Mix | A25742 | Thermo Fisher Scientific | USA |
| Reverse Transcription Kit | RR036A | TaKaRa | JAPAN |
| si-GAS5 | GZP22062100008 | Tsingke Biotechnology | China |
| miR-223-3p mimic/inhibitor/NC | miR10000665 | RiboBio | China |
| Firefly luciferase and renilla luminescence luciferase activities | RG027 | Beyotime | China |
| Hematoxylin and Eosin Staining Kit | C0105S | Beyotime | China |
